# An impaired natriuretic peptide hormone system may play a role in COVID‐19 severity in vulnerable populations

**DOI:** 10.1096/fba.2020-00042

**Published:** 2020-09-10

**Authors:** Mark G. Currie, Daniel P. Zimmer, Perry V. Halushka

**Affiliations:** ^1^ Cyclerion Therapeutics Cambridge MA USA; ^2^ Medical University of South Carolina Charleston SC USA

## Abstract

Advanced age, underlying cardiovascular disease (including hypertension), and obesity are associated with a higher risk of progression to severe hypoxemia, acute respiratory distress syndrome (ARDS), and death in COVID‐19‐infected patients. African Americans have a higher degree of COVID‐19 mortality. The incidence of salt‐sensitive hypertension is higher in older individuals and African Americans. Lower circulating levels of natriuretic peptides, key regulators of vascular tone and kidney function, have been associated with salt‐sensitive hypertension and obesity. Evidence has accumulated that ANP administered to pulmonary endothelial cells, isolated lungs, and patients suffering from ARDS reduces endothelial damage and preserves the endothelial barrier, thereby reducing pulmonary edema and inflammation. Epidemiologic and pharmacologic data suggest that deficiencies in the natriuretic peptide hormone system may contribute to the development of severe lung pathology in COVID‐19 patients, and treatments that augment natriuretic peptide signaling may have potential to limit progression to ARDS.

**Abbreviations**
ANP: atrial natriuretic peptide
ARDS: acute respiratory distress syndrome (ARDS)
BNP: brain natriuretic peptide
cGMP: cyclic guanosine 3’ 5'‐monophosphate
IQR: interquartile range
LPS: lipopolysaccharide
NT‐proBNP: N‐terminal pro‐brain natriuretic peptide
TNFα: tumor necrosis factor α

## 1 INTRODUCTION

### 1.1 The need for novel treatments for covid‐19‐infected patients based on pharmacologic and biochemical data

There is an urgent need for treatments for COVID‐19‐infected patients, particularly for the most vulnerable populations. Prevention or early treatment of acute respiratory distress syndrome (ARDS), the main cause of death in COVID‐19‐infected patients may have a significant impact on their clinical course.

Several studies have reported a higher prevalence of cardiovascular disease including hypertension, obesity, and diabetes in hospitalized patients with COVID‐19, and there is evidence that these comorbidities are associated with disease progression to hypoxemia, ARDS, and death.^1^ Hypertension has emerged as one of the specified risk factors. Hypertension is a complex disease with many different genes and environmental factors that contribute to its development.^2^

It has been reported that in the United States, there has been a disproportionately high rate of COVID‐19‐related hospitalizations and death among older individuals and African Americans.^1^, ^3^, ^4^ This potentially provides a clue to genetic and biochemical factors that may be involved in the sensitivity of these patients with cardiovascular disease to COVID‐19.

Hypertension is more prevalent in older individuals and African Americans than in in non‐Hispanic white men and women (age‐adjusted prevalence of 44.4% and 43.9% in Black men and women, respectively, vs 34.1% and 30.3% in non‐Hispanic white men and women). Furthermore, both populations have a high degree of salt sensitivity and salt‐sensitive hypertension.^5^ The pathobiology underlying hypertension and salt sensitivity in these populations may also be implicated in their vulnerability to COVID‐19‐induced ARDS.

One key factor that has been associated with salt sensitivity is impaired secretion of atrial natriuretic peptide (ANP). ANP is a key hormone in the regulation of vascular tone and integrity, kidney function, and blood pressure^6^; there is clear evidence that impairment of this system contributes to hypertension, especially salt‐sensitive hypertension.^5^, ^7^ Plasma ANP levels, which increase in normotensive subjects fed a high‐salt diet, paradoxically decrease in Black hypertensive subjects in response to a high‐salt diet ^8^. In 3148 individuals free of prevalent cardiovascular disease enrolled in the Dallas Heart Study, hypertension was present in 41%, 25%, and 16% of Black, white, and Hispanic individuals, respectively, while unadjusted N‐terminal pro‐brain natriuretic peptide (NT‐proBNP) levels were lower in Black individuals (median: 24 pg/mL; interquartile range [IQR]: 10 to 52 pg/ml) than in white (32 pg/mL; IQR: 16 to 62 pg/mL), *P* < .0001) and Hispanic individuals (30 pg/mL; IQR: 14 to 59 pg/mL).^9^ Similarly, levels of NT‐proBNP, a precursor to BNP, were significantly lower in African Americans (43 pg/mL; IQR: 18 to 88 pg/mL) than Caucasians (68 pg/mL; IQR: 36 to 124 pg/mL; *P* < .0001) in a large cohort study.^10^

This striking relationship between COVID‐19 disease severity and natriuretic peptide levels can also be found in obese individuals. It is well established from epidemiological studies that circulating natriuretic peptide levels are lower in obese individuals.^11^, ^12^, ^13^ Several studies have found that obese individuals are more likely to develop severe complications of COVID‐19.^14^, ^15^

The natriuretic peptides ANP and BNP are best known for their roles in the cardiovascular system: they were first identified as peptide hormones secreted by the heart. As agonists of the guanylate cyclase A receptor, both ANP and BNP increase cyclic guanosine 3’ 5'‐monophosphate (cGMP); and their pharmacologic effects on the vasculature (vasodilation) and kidney (natriuresis) have been well documented.^16^ Furthermore, BNP and its precursor NT‐proBNP are routine clinical laboratory measures used as prognostic markers in cardiology. NT‐proBNP has been shown to be elevated in COVID‐19 patients with severe disease and non‐survivors, most likely an indication of increased cardiac stress.^17^, ^18^, ^19^

What may be underappreciated is the accumulated evidence that the natriuretic peptides also play an important protective role in the lungs. In the late 1980s, ANP was discovered to block thrombin‐induced increases in endothelial permeability.^20^, ^21^ This research was extended by multiple groups to show that the ANP‐attenuated increases in lung endothelial permeability caused by various insults, including oxidant stress, lipopolysaccharide (LPS), and tumor necrosis factor α (TNFα).^22^, ^23^ ANP reduced secretion of inflammatory mediators in response to LPS in macrophages,^24^ and stimulation of the cGMP pathway suppressed TNFα‐mediated increases in levels of endothelial and leukocyte‐derived soluble adhesion molecules in a mouse model of inflammation.^25^ The lung‐protective effects of ANP infusion were demonstrated in animal studies. ANP pretreatment protected mice from endothelial barrier dysfunction and inflammation induced by LPS or Staphylococcus aureus infection.^26^ Finally, a clinical study in patients with acute lung injury provided the first demonstration of the therapeutic potential of ANP in ARDS. ANP infusion improved arterial oxygenation and lung injury score in patients with acute lung injury during mechanical ventilation.^27^ The importance of targeting the endothelium in COVID‐19 is underscored by the fact that lungs from COVID‐19 patients exhibit severe vascular endothelial injury.^28^ Lending support to the therapeutic hypothesis of cGMP modulation in COVID‐19, an approach using inhaled nitric oxide (NO) is currently under investigation in 9 clinical trials in COVID‐19 patients (clinical trials.gov: NCT04383002, NCT04338828, NCT04358588, NCT04305457, NCT04305457, NCT04337918, NCT04306393, NCT04421508, NCT04398290).

## 2 CONCLUSION

Epidemiologic and pharmacologic data suggest that COVID‐19‐infected patients who have deficiencies in the lung‐protective natriuretic peptide hormone system are especially vulnerable to development of severe lung complications when infected with COVID‐19. Given the lung‐protective effects of natriuretic peptides, we suggest investigation of treatments that directly affect the natriuretic peptide‐cGMP signaling pathway in COVID‐19 patients. Such an approach may prevent COVID‐19‐induced increases in lung permeability, inflammation, and subsequent development of ARDS and the need for ventilator therapy. Natriuretic and diuretic effects of these treatments may also limit pulmonary edema and protect the kidney from injury, a complication that develops in patients with severe disease.^29^, ^30^ There is also evidence that a natriuretic peptide treatment approach has the potential to attenuate the coagulopathy associated with COVID‐19,^30^, ^31^, ^32^ and the cardioprotective effects of natriuretic peptides^33^ may attenuate COVID‐19 myocarditis and acute cardiovascular syndrome.^34^, ^35^ Limiting progression of the disease to this stage could have a profound impact on number of deaths since most patients who require mechanical ventilation die (88.1% in NYC cohort,^1^ 93% in Wuhan study^30^). This treatment approach may be most beneficial for COVID‐19 patients who have dysregulated natriuretic peptide signaling, including African Americans, obese patients, and elderly patients, who appear to be most vulnerable to COVID‐19.

We envision two potential therapeutic approaches to treating COVID‐19‐infected patients that take advantage of the natriuretic peptide pathway. The first potential investigative approach is an oral treatment using a neutral endopeptidase inhibitor, such as sacubitril, which has been shown in clinical studies to raise endogenous levels of ANP and BNP.^36^, ^37^ We posit that sacubitril could be given orally early in the presentation of patients with COVID‐19. A second exploratory approach would involve administration of an IV infusion of ANP (carperitide) or BNP (nesiritide) in hospitalized patients before they have advanced to ARDS. We believe that either or both approaches could have a profound positive effect on the progression of the disease, decrease the need for intensive treatment with ventilators, and reduce morbidity and mortality in COVID‐19‐infected patients. Each approach may have unique advantages: an inhibitor approach using sacubitril is expected to raise C‐type natriuretic peptide levels, providing greater vasculoprotection,^38^ and an agonist approach using nesiritide or carperitide does not rely on and therefore is not limited by endogenous levels of the natriuretic hormones. As there are drugs with these mechanisms of action available in the US for use in other indications, such drugs may support rapid investigation of such therapeutic approaches in an appropriately controlled setting.

## CONFLICT OF INTERESTS

MGC is employed by and owns stock and options in Cyclerion Therapeutics. In addition, Dr. Currie has a patent EARLY DRUG INTERVENTIONS TO REDUCE COVID‐19 RELATED RESPIRATORY DISTRESS, NEED FOR VENTILATOR ASSISTANCE AND DEATH pending. DPZ is a paid consultant for and owns stock in Cyclerion Therapeutics. PVH has no conflict of interest.

## AUTHOR CONTRIBUTIONS

Mark G. Currie conceived the hypothesis. Mark G. Currie, Daniel P. Zimmer, and Perry V. Halushka all contributed to the writing and editing of this manuscript.
